# Knowledge boundaries for implementation of quality improvement interventions; a qualitative study

**DOI:** 10.3389/frhs.2024.1294299

**Published:** 2024-06-11

**Authors:** Hilda Bø Lyng, Torunn Strømme, Eline Ree, Terese Johannessen, Siri Wiig

**Affiliations:** ^1^ SHARE—Centre for Resilience in Healthcare, Faculty of Health Sciences, University of Stavanger, Stavanger, Norway; ^2^ Department of Health and Nursing Sciences, Faculty of Health and Sports Science, University of Agder, Kristiansand, Norway

**Keywords:** knowledge boundaries, implementation, quality improvement, interventions, nursing homes, homecare services

## Abstract

**Introduction:**

Implementation and adoption of quality improvement interventions have proved difficult, even in situations where all participants recognise the relevance and benefits of the intervention. One way to describe difficulties in implementing new quality improvement interventions is to explore different types of knowledge boundaries, more specifically the syntactic, semantic and pragmatic boundaries, influencing the implementation process. As such, this study aims to identify and understand knowledge boundaries for implementation processes in nursing homes and homecare services.

**Methods:**

An exploratory qualitative methodology was used for this study. The empirical data, including individual interviews (*n* = 10) and focus group interviews (*n* = 10) with leaders and development nurses, stem from an externally driven leadership intervention and a supplementary tracer project entailing an internally driven intervention. Both implementations took place in Norwegian nursing homes and homecare services. The empirical data was inductively analysed in accordance with grounded theory.

**Results:**

The findings showed that the syntactic boundary included boundaries like the lack of meeting arenas, and lack of knowledge transfer and continuity in learning. Furthermore, the syntactic boundary was mostly related to the dissemination and training of staff across the organisation. The semantic boundary consisted of boundaries such as ambiguity, lack of perceived impact for practice and lack of appropriate knowledge. This boundary mostly related to uncertainty of the facilitator role. The pragmatic boundary included boundaries related to a lack of ownership, resistance, feeling unsecure, workload, different perspectives and a lack of support and focus, reflecting a change of practices.

**Discussion:**

This study provides potential solutions for traversing different knowledge boundaries and a framework for understanding knowledge boundaries related to the implementation of quality interventions.

## 1 Introduction

The implementation of interventions aiming to improve quality of care is in everyone's interest. However, the implementation and adoption of new ways of working have proved difficult, even in situations where all participants recognise the benefits of the intervention (1). Healthcare is characterised by clinical domain specialists working within their silos, performing specialised knowledge intensive practices (2, 3). This specialisation introduces cognitive, physical and cultural boundaries between individuals and groups. Closing these boundaries requires efforts for converging of perspectives, knowledge and interests in order to develop shared understanding and objectives.

One way to describe difficulties in implementing new interventions is to explore different types of boundaries for transfer and adoption of new knowledge. Carlile (4) describes three different knowledge boundaries, to be traversed in his integrated framework: (1) syntactic, (2) semantic, and (3) pragmatic boundaries. Furthermore, Edenius et al. (2) found these knowledge boundaries relevant for describing knowledge boundaries during implementation of innovations in healthcare settings.

The syntactic boundary refers to situations with challenges in the processing of knowledge between sender and receiver (2, 4–6). As such, this boundary focuses on challenges in the transfer of knowledge, and not on difficulties in understanding the knowledge. At the syntactic boundary, knowledge is perceived as explicit and not as causing problems for shared understanding. In order to traverse the syntactic boundary, the amount of knowledge to be transferred needs to be optimised. Such optimisation can be achieved by either increasing the recipient's ability to absorb knowledge (absorptive capacity) (7) or by using a more efficient and suitable channel for the knowledge transfer (4, 8).

Characteristics for the semantic boundary are a lack of shared understanding, causing misunderstandings among actors (4, 9). As such, challenges are now shifted from improving absorptive capacity and optimising transferring channels to the knowledge itself. Knowledge and language are highly contextual and will therefore vary across disciplines, roles, and organisations (5, 10). Facilitating factors for traversing the semantic boundary are to provide some form of knowledge translation, like the use of boundary objects [defined as “an object that lives in multiple social worlds and which has different identities in each” (11)], visualisations, or through the development of a shared vocabulary (4, 12–14) as well as through brokers/intermediaries/boundary spanners (who “facilitate the communication and sharing of expertise, linking groups who might be separate in terms of location, division, or function” (3, 15–17).

The third knowledge boundary is called the pragmatic boundary and relates to changing of practices (2, 4, 9), meaning that this boundary refers to differences in incentives, perspectives, and interests. Translation activities can no longer bridge the actors involved, and a transformation of knowledge is needed to traverse this boundary (4, 18). Transforming knowledge is not an easy task, as it requires a change of practices and routines. The way we have invested our situated knowledge into practice takes time, and changing the way we do things might therefor be perceived as putting your expertise at stake. Traversing the pragmatic boundary therefore relies on motivation and willingness to align interests, perspectives, and incentives (9).

Healthcare practices are knowledge intensive, and changing these practices is therefore often met with some form of resistance (1, 2). As such, interventions to improve quality in healthcare need to provide a good reason and motivation for participants to be willing to change their situated knowledge and practices (2, 19). Due to the high level of specialisation, occupational communities form a local understanding of their work and practices (5, 20).

The empirical background for this study is the implementation of two different quality improvement interventions in Norwegian nursing homes and homecare services. The first intervention (external case) concerned a novel researcher-developed leadership intervention, where the implementation process was driven externally by the researchers involved. The second intervention (internal case) focused on practices for recognising deterioration in patients. This intervention followed an internally driven implementation process where the organisation itself was responsible for selecting which intervention to implement and for the implementation process. This study aims to identify knowledge boundaries influencing the implementation processes in both these cases.

Healthcare is becoming more and more knowledge-intensive and quality of healthcare services therefore relies on implementation of new evidence-based knowledge and for healthcare actors to update the situated knowledge and practices to new knowledge (21). Traditional research on implementation has provided valuable knowledge on approaches and barriers and how to best succeed with the implementation process. However, there is also a need to take the type of knowledge to be implemented into account, as quality interventions and guidelines are not “knowledge objects” that can be transferred across organisations without facing knowledge boundaries (21, 22). By looking into diverse types of interventions we will develop new understanding of knowledge boundaries influencing implementation processes that is of relevance for clinical practice and leadership roles in healthcare. As such, this study responds to calls for exploring different types of interventions in order to provide an understanding of different contextual aspects of implementation processes (19, 23, 24).

The following research question (RQ) guided the study: What type of knowledge boundaries can be identified in quality improvement intervention processes?

## 2 Materials and methods

A qualitative methodology was chosen based on the exploratory nature of the research questions. The empirical data stems from the project: “Improving Quality and Safety in Primary Care—Implementing a Leadership Intervention in Nursing Homes and Homecare” (SAFE-LEAD) (2016–2023). The SAFE-LEAD project is a multiple case study whereby the implementation of two different improvement interventions took place in Norwegian nursing homes and homecare services. The externally driven implementation of a leadership intervention makes up the largest case, and was supplemented with a tracer project engaged in an internally driven quality intervention (25, 26). The theoretical sampling of sites was based on variety in size and geographical location, to best illustrate the Norwegian setting (rural areas, medium sized, large cities and city areas) (27). Each case had their own research aims but the overall project aimed to explore differences in the implementation process across cases, including identifying enablers and barriers for different types of implementation approaches. This study specifically provides understanding of cross-case barriers. Combining externally and internally driven implementation processes in this study allowed for a rich empirical foundation for understanding knowledge boundaries in relation to different implementation processes for quality improvement interventions across nursing homes and homecare services. Contextual setting.

In Norway, nursing homes and homecare services (like other primary care services such as general practitioners) are the responsibility of municipalities (28, 29). There is a regulation in place to ensure continuous improvement of quality and safety in primary care to guide leaders in their work. The regulation applies requirements for leaders to plan, implement, and evaluate quality improvement interventions in their organisations. Leaders are therefore key actors for quality and improvement work in nursing homes and homecare services. Based on data from the SAFE-LEAD study, this study focuses on leaders as informants to explore implementations of quality interventions. The two cases included in this article reflect different approaches of implementation processes, thereby enriching the empirical foundation of this study. However, both cases share strong similarities as both focus on implementing quality improvement interventions in primary care, both use leaders as informants, and both are within the Norwegian setting.

### 2.1 External case

The external case reports from the implementation of a novel researcher-developed quality intervention for primary care leaders (26, 30). The intervention included a leadership guide aimed to support leaders in their quality and safety work. Specifically, the intervention entailed three steps following the structure of the leadership guide, with associated researcher-facilitated workshops. The participants received unsupervised “homework” to be performed in between the workshops. The first workshop focused on identifying situational challenges for quality within their own setting. The following “homework” required leaders to evaluate and score their organisation using the scoring tool provided from the leadership guide. The second workshop focused on developing improvement objectives for the challenges identified from the first step. The second “homework” requested the participants to develop and describe formalised objectives for their organisation. In the third workshop the leaders worked on developing action plans to achieve their formalised objectives. The third round of “homework” requested the leaders to translate their described action plans into practice. The researcher's role in the implementation process was to facilitate all workshops (presentations, discussions, and “homework” reviews). The participants encompassed leaders from 8 different units (4 nursing homes and 4 homecare services), located within 4 municipalities (one rural, one medium-sized, a large city and a large city area). There were 12 interviews (3 focus groups (*n* = 15) + 2 individual interview pre intervention, 4 focus group interview midway (*n* = 23) and 3 focus group (*n* = 16) post intervention) during a 1-year intervention process where the same units (with the same leaders participating in interviews) make up the longitudinal empirical data for the external case. The variety of participants for the pre, midway, and post interviews reflects availability of leaders to take part. The interviews lasted between 60 and 90 min. Participating sites were recruited by municipal services, and site managers further recruited a management team (unit managers, department managers, professional development nurses, coordinator, system officer) to participate in the implementation process (30). Data collection for the external case was performed by authors SW, ER, TJ and TS and other researchers in the project group. All participants signed consent forms.

### 2.2 Internal case

The internal case reports from an internally driven implementation process in homecare. The chosen intervention for implementation concerned observational competence improvement (31, 32). The intervention focused on formal teaching of new knowledge for observation of patients, training of new skills, simulation training of new procedures and measurements, and the introduction of new equipment. The formal teaching was organised by the county's Centre for Development of Institutional and Homecare Services (DIHS), while the remainder was organised by and within the different homecare organisations included (31, 32). DIHS provided the researchers with contact to two different homecare districts, one urban and one mixed urban/rural (HBT1 and HBT 2), that had decided to implement this specific quality improvement intervention. The empirical data for this article includes interviews with leaders (homecare leaders and development nurses) after the implementation period was completed to obtain understanding of leaders' experiences and evaluations of the implementation process. Consent forms were collected from all participants. Only interviews of leaders (responsible for a homecare district) and development nurses (*n* = 8) were used in this study to allow for validity in the cross-case analysis. The semi structured interviews lasted about an hour and totalled 100 pages of transcripts. Data collection for the internal case was performed by TS over a period of two months in 2020 after completing a one-year intervention. The was no overlap of participants across the cases.

### 2.3 Data analysis

**Figure 1:**
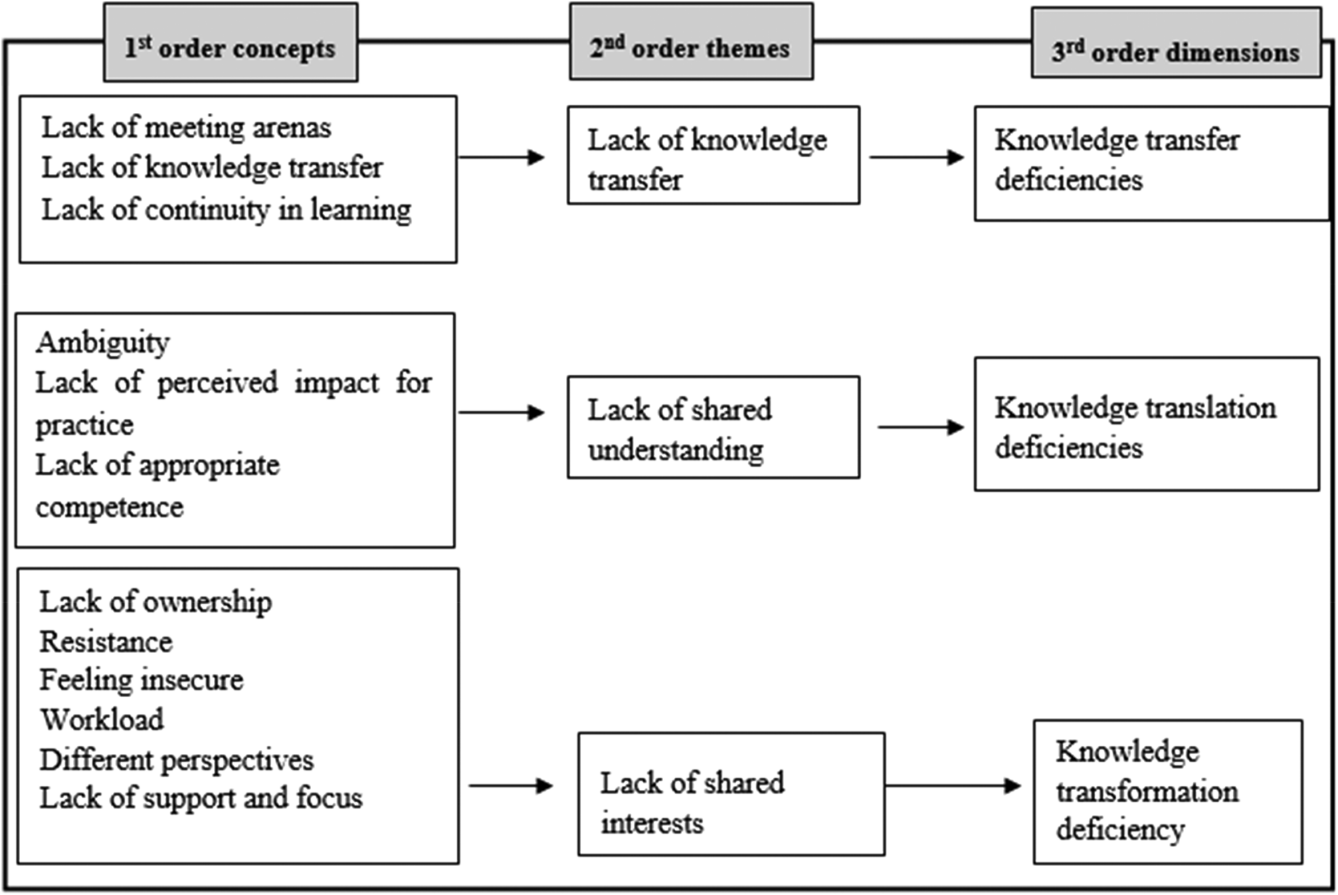
Data structure model.

All interviews were audio recorded and transcribed verbatim. The analytical process was a three-step process. The explorative nature of RQ1 informed our choice of first using inductive grounded theory (33, 34) to analyse the data. The initial aim of this study was to develop new theoretical understanding on barriers for implementation of new knowledge. Grounded theory is a recommended method to address research questions of implementation processes and for theory development (27, 35). As such, we could explore and identify boundaries and themes emerging directly from the data. The literature was not reviewed before the data analysis and no framework was chosen for guiding the analysis in line with grounded theory methodology (27). The empirical data from each case was analysed individually following the framework of Gioia et al. (33) of identifying 1st order codes directly from the data, and then aggregating into 2nd order themes and 3rd order dimensions, see Figure 1. Author HBL led the inductive analysis and consensus from all authors on the aggregation into themes and dimensions. The analysis was performed in the NVivo 1.7 software. The internal case resulted in 1,845 references distributed on 284 different 1st order codes. The external case entailed 3,286 references distributed on 190 1st order codes.

In the second step, all 1st order codes related to knowledge deficiencies (3rd order dimensions), entailing 56 out of 284 1st order codes for the internal case and 44 out of 190 1st order codes for the external case, for implementing quality improvement interventions were combined across cases in a constant comparison process (27). Consensus was formed by all authors. In this process, inductive 1st order codes with similar meaning and content were merged, despite some 1st order codes having slightly different inductive names like “lack of meeting arenas”/“lack of arenas”/“lack of learning arenas” or “lack of perceived impact for practice”/“lack of relevance” if they shared the same inherent meaning.

The third step introduced a new deductive thematic analysis (36) of the findings from the first and second steps, due to the discovery that the results shared relatedness to the knowledge boundaries framework by Carlile (4). The findings (knowledge deficiencies) were in this step grouped into syntactic, semantic, and pragmatic boundaries by author HBL. Most of the 1st order codes of knowledge deficiencies included aligned well with the knowledge boundaries framework by Carlile (4). Due to the aim of the study and the focus towards identifying the most influential boundaries and not outliers, a few identified knowledge boundaries were not included in the resulting framework of this analysis (disrespectfulness, too many actors involved, lack of confidence, cannot pause the process, and sessions being too long).

## 3 Results

In terms of identifying knowledge boundaries for implementation of quality interventions, findings from the inductive first and second steps of the analysis are illustrated in the data structure model, see Figure 1.

In accordance with the third analytical step, in which the findings were related to syntactic, semantic, and pragmatic knowledge boundaries, the following results section will describe each knowledge boundary with the associated inductive codes.

### 3.1 Knowledge transfer deficiencies/syntactic boundary

Knowledge boundaries related to knowledge transfer deficiencies included a lack of meeting arenas, lack of knowledge transfer, and lack of continuity in learning. Each of these concepts will be described in more detail in the following.

#### 3.1.1 Lack of meeting arenas

Lack of meeting arenas was reported as a barrier in both implementation cases. In order for knowledge transfer, learning and adoption to take place, some arenas where the healthcare workers can meet up need to be available. Lack of arenas was found to hamper knowledge transfer and learning, and furthermore to make it more difficult to disseminate information to staff simultaneously, as their hectic work schedule provided few opportunities for face-to-face information transfer and reflection. This can be exemplified in the following quote, where a regular meeting arena was cancelled due to the Covid-19 pandemic regulations. Lack of meeting arenas refer to the need for efficient knowledge transfer channels, which is a characteristic for transversing the syntactic boundary.

*“Before Covid we have used these ‘blackboard-meetings’. And then (after Covid) we found that we then lost a really important arena for reflection. The staff has now started to request these meetings themselves, because there is information that should have been communicated to all teams. We have 3 teams here, and all of them should receive the same information”*. (Internal case, Site HBT 1, Informant 2).

#### 3.1.2 Lack of knowledge transfer

Having meeting arenas available is not enough in itself, if these arenas are not used appropriately for knowledge transfer and learning activities. Knowledge transfer is more than just having arenas, since it also includes choosing an appropriate channel for transferring the knowledge. A challenge reported in both cases was to ensure transfer of knowledge to all staff, who might be working different shifts, with many in part-time positions. Channels for knowledge transfer therefore need to be aligned with the type of knowledge to be transferred (e.g., practices need face-to-face meetings), and channels used (e.g., staff meetings or email). The following quote, illustrates that sending a few employees on a course, did not necessarily result in the dissemination of this acquired knowledge across the organisation. This finding also confirms the need to establish efficient channels for knowledge transfer, to traverse the syntactic boundary.

“*We find it difficult to anchor it. We are sending 1 or 2 employees to a course, and they come back to inform the others of what they learned. But we would have benefitted more by having the course internally… We somehow can’t figure out how to transfer knowledge to the districts (different homecare teams)*”. (External case, Site E, Informant 2).

#### 3.1.3 Lack of continuity in learning

To increase the adoption of knowledge among staff, findings showed that it was important to provide continuity in the learning activities. Introducing new knowledge and practices in a single event (1–2 day course) did not have the same impact as repeated focus on internalising (e.g., weekly simulation training) new knowledge and practices. This points to the need for sustained focus, learning and training.

“*I found the course day very interesting. But it’s a bit like, you get there, become inspired, and then leave again. So, after some time you have actually forgot about the course day. So, I would like to have a more continuous focus*” (Internal case, Site HBT 1, Informant 3).

### 3.2 Knowledge translation deficiencies/semantic boundary

#### 3.2.1 Ambiguity

Developing an understanding of the intervention was key to the implementation process, and particularly for the facilitators (actors with a key role in driving the implementation process within the nursing homes or homecare services) who oversaw the training and homework activities. However, some facilitators felt that they needed more training in the practices included in the intervention, before having to facilitate training and learning for others. Perceiving the content of the intervention as ambiguous, reduced their motivation for taking on such responsibilities. This finding reflected a lack of shared understanding between intervention providers and facilitators within the organisation, which is described as a characteristic of the semantic boundary.

*“And suddenly I was in charge of the simulation, without knowing what the exact content of it was. I found this quite hard. I didn’t want this responsibility at all”*. (Internal case, Site HBT 1, Informant 2).

#### 3.2.2 Lack of perceived impact for practice

The willingness to devote the necessary time and resources to adopting new knowledge and practices relied on participants and facilitators perceiving the intervention as having a desired impact on practice. It was therefore crucial for the facilitators to clearly convey the rewards of the intervention to the participants. If the perceived impact for practice was missing, the participants' incentives for engagement were reduced. This finding relates to a lack of shared understanding among participants and facilitators, as in the following quote, where the facilitator perceived the intervention as too general and too comprehensive, thus introducing a semantic boundary for the implementation process.

*“I have informed about it (the intervention) in staff meetings. But I don’t think they see the impact of it (the intervention). I don’t think so. And to me, this way of working is a bit difficult. However, that might be caused by me being located in a different building. To me, this (the intervention) was a bit too general and too comprehensive”* (External case, Site D, Informant 5).

#### 3.2.3 Lack of appropriate competence

The need to provide facilitators with appropriate knowledge, experience, and training to make them feel secure in their role was found important for both cases. Many facilitators felt insecure in performing the tasks they were put in charge of because they lacked either the appropriate background knowledge or the appropriate experience in elements of the intervention to be able to fully understand what they were to teach others. This finding also reports a lack of shared understanding between intervention providers and facilitators.

*“I don’t totally understand what this (the intervention) is. I find it a bit difficult, but I have also reported back that it has been difficult and that I haven’t received appropriate professional support to do this. Because it is not enough to be provided with some material (slides), developed by others, when you have not previously worked with these things yourself. Standing in front of the others and being trustworthy is therefore difficult”*. (Internal case, Site HBT 2, Informant 2).

### 3.3 Knowledge transformation deficiencies/pragmatic boundary

#### 3.3.1 Lack of ownership

A typical challenge at the pragmatic boundary was that the introduction of new practices made healthcare actors take on unfamiliar practices, thereby turning experts into novices. In the internal case, some managers who normally engaged with administrative work, had to teach their staff practices they had not performed themselves for many years, as exemplified in the quote below. However, as also reflected on in the quote, feeling unfamiliar with training others did not necessarily end up with a lack of ownership in the long run. When the result turned out successful, as reported in the quote, people still developed motivation for further engagement. The difference between interventions being perceived as too unfamiliar and the ability to cope with the unfamiliarity was a balancing act, however, and varied across the facilitators involved. This finding reflected the lack of shared perspectives.

*“We were having facilitator training. And then they put me in charge of teaching VAP (venous access port) to the others. I have not touched VAP in many years. The person who was supposed to take this responsibility vanished out in thin air; I don’t know where she was off to. And then I was left with the responsibility of training the others, and I thought that this is not OK. However, it all went very well”*. (Internal case, Site HBT1, Informant 3).

The facilitation of both interventions required a lot of organisational efforts to plan for teaching, meetings, discussions, and training. Investing the necessary time and resources in the organisation of the intervention relied on ownership and shared interests between facilitators; if not, it easily ended up as half-hearted attempts.

*“The intervention was not concrete enough and I had maybe not enough ownership of it (the intervention). We discussed this at the start-up, in what way we were to organise this project. Whom to include, and whom to exclude. We ended up with including too many people”*. (External case, Site D, Informant 5).

#### 3.3.2 Resistance

Resistance was found to act as a boundary to implementation in our study. The intervention facilitators experienced some participants being against all types of changes introduced, as described in the first quote below. Furthermore, resistance could also be culture related, where some healthcare workers continued to perform their work as they usually did, despite receiving new knowledge and practices, as described in the second quote below. As such, resistance reflects the difficulty of changing practices and a lack of shared interests, as described as part of the pragmatic boundary.

*“I have received feedback that changes go quite well when they (staff) receive enough information. However, there are always some that still find all type of changes horrible”*. (External case, Site D, Informant 1)

*“The intervention has provided increased levels of knowledge within the department and an increased focus on professional work, yet at the same time there has not been an increase in the number of professional discussions. That surprises me. The few nurses that seek to initiate a professional discussion are quickly silenced… Even though they now get tools and knowledge of what to look for. And then it is not used. It’s quite surprising”*. (Internal case, HBT 1, Informant 1).

#### 3.3.3 Feeling insecure

As described above, the introduction of new knowledge and practices was perceived challenging in both cases. As a result, some facilitators felt insecure about their tasks and role, as they questioned their situated competence to perform what was expected of them. This finding reflects the aspect of feeling like a novice when performing new practices as part of the pragmatic boundary.

*“There and then I felt that they expected more of me than I could be, do you understand? I don’t know how much and what type of knowledge they thought I had. It has been many years since I worked clinically. And then I have had cancer, meaning that I have put my focus elsewhere. And suddenly I’m in the middle of something and thinking, gosh, I can’t do this? What do I do? It was so comprehensive. I didn’t know how I could read up on this on my own. And when I asked for supervision, it turned out that I was supposed to be a supervisor for others”* (Internal case, Site HBT 1, Informant 3).

Some leaders also felt insecure on behalf of their staff. The time set aside for learning new knowledge and practices was limited, and the leaders sometimes asked themselves whether it was safe to send staff to the patient's home to independently perform new practices. This finding also reflects a lack of shared perspective described as part of the pragmatic boundary.

*“It has been challenging to have so many people in training. It affects the whole department. Because everyone is are suddenly to be involved in the training. And then it must be fairly quick. It didn’t feel good. We had 30 people in for the 1-hour medication administration training. And they have not been handling medications before, any of them. And they have not participated in the medication courses before. This makes me start thinking that these people will be sent out on their own to patients, who can become very ill”* (Internal case, Site HBT1, Informant 3).

#### 3.3.4 Workload

Workload acted as a marked barrier to the implementation process in both cases. The workload barrier concerned situations with limited resources to meet the work demands and unpredictability in their working day imposing a need for re-prioritisation. This unpredictability and lack of resources were found to hamper the facilitation of the implementation process. Facilitators were sometimes frustrated about continuously having to alter their plans, which for them meant additional work as described in the quote.

“*It concerns prioritisations. I have sometimes been thinking that we need to fight for more time to use in this project (intervention). We plan for it, get an overview of people at work, and decide on a participating group. And then something happens. And patients will always be the most important”* (Internal case, HBT 2, Informant 4).

Workload was also reported as a key barrier to the internalisation of the intervention. Healthcare workers and facilitators involved in the interventions often had to pause their work on the intervention and instead attend to more pressing matters. This on and off pattern hampered the process, as described below.

*“We want everything to be done well. And to do so we need to prioritise and find resources. So, if we could have had more time for systematic meetings, we would have worked much more with these things and ensured more involvement. Yet, day-to-day operations go on, and the hours at work fly by, and there are so many tasks to do, and not so much time available compared to the number of tasks. We therefore constantly need to set priorities. And when we pick it (the intervention) up again, we have to start over again, and reset ourselves, because we can’t remember were we left it (the intervention) last time”* (External case, Site E, Informant 2)

#### 3.3.5 Different perspectives

Having different perspectives is a characteristic of the pragmatic boundary. Different perspectives were found regarding how to use new knowledge and practices in everyday work. Some healthcare workers included the newly introduced practices in every consultation while others continued working as before, pointing to different perspectives on what their job entailed and the need for continuous improvement in healthcare positions.

*“When it comes to everyday practices, like measuring the blood pressure at peaceful times, and establishing a status, it is up to the individual healthcare workers to decide. Not everyone takes responsibility. We are a big group and there are differences in how people think about their job, and also their reason for why they forget things. Working in homecare services is often also unpredictable and chaotic by nature”* (Internal case, Site HBT 2, Informant 4).

#### 3.3.6 Lack of support and focus

The internalisation of new knowledge and practices was found to rely on continuous focus and facilitators' support of staff. It was not a one-off event to teach new knowledge and practices, but instead a continued focus on including the new knowledge and practices in everyday work and their organisational culture. As such, continuity of facilitators and leaders was essential for maintaining support and focus as described in the quote.

*“It (the intervention) has been fun, and we received some incentives to do this (the intervention). It is therefore a shame that our leader has left. We have lost the person who had the overall responsibility. And then it (the intervention) is drowning a bit in everything else. But I think we got some good incentives. It will be exciting to see what comes out of this in the end”* (External case, Site D, Informant 3).

## 4 Discussion

Our findings show that boundaries identified for implementing quality interventions can purposefully be grouped into the framework of Carlile (4) for syntactic, semantic, and pragmatic knowledge boundaries. 1st order codes making up the different knowledge boundaries are displayed in the middle row as *Challenges* in Table 1. In line with Edenius et al. (2) in the discussion we will explore how the different knowledge boundaries relate to different situational circumstances, and discuss potential solutions in terms of existing theory.

**Table 1:** Knowledge boundaries in terms of circumstances, challenges, and potential solutions.

	Syntactic boundary	Semantic boundary	Pragmatic boundary
Circumstances	The syntactic boundary is mostly related to the dissemination and training of new knowledge and practices to staff.	The semantic boundary is mostly related to perceived uncertainty in the facilitator role.	The pragmatic boundary is mostly related to the change in practices and to an unsecure feeling in doing so.
Challenges	Lack of meeting arenas	Ambiguity	Lack of ownership
	Lack of knowledge transfer	Lack of perceived impact for practice	Resistance
			Feeling insecure
	Lack of continuity in learning	Lack of appropriate competence	Workload
			Different perspectives
			Lack of support and focus
Potential solutions	To ensure meeting arenas and regular meeting agendas.	To provide facilitators with a deep understanding of the relevance of the intervention.	To support staff and facilitators in their work of training and internalising new knowledge and practices.
	To provide continuity in learning activities to ensure increased familiarity and internalisation.		
			To develop a culture for change, thus easing the ability for traversing the pragmatic boundary.
		To ensure translation of tacit knowledge (practice-based) into explicit knowledge for learning new practices.	
			To align the implementation process to their workload.
	Knowledge that is common (obvious) should be transferred through the most efficient and effective media.		
		Knowledge that is unclear should inform facilitators to introduce means for translation and boundary objects.	Knowledge that needs negotiation should inform facilitators to develop shared objectives, understanding of different interests, and use of boundary objects.

The syntactic boundary included boundaries like the lack of meeting arenas, lack of knowledge transfer, and lack of continuity in learning. The syntactic boundaries were found to be mostly related to challenges of dissemination and training of new knowledge and practices for all staff across the organisation. Healthcare workers in nursing homes and homecare services are a group with a great variety of training and education (from specialised nurses with Master's degrees, to health workers without formal training), and expertise (some have worked for decades and others are quite new to the setting; some are working full time, and others only work some shifts along with studies). As such, these healthcare workers require different training and learning activities. The dissemination of new knowledge and practices across staff was therefore found difficult, even in situations where the knowledge to be transferred was not in need of translation or transformation, due to challenges in finding appropriate arenas and means for the transfer. Providing appropriate and regular meeting places for knowledge transfer are therefore a potential solution for circumventing the syntactic boundary. Challenges at the syntactic boundary refer to the identification of ways to optimise the amount of knowledge being transferred, increasing the absorptive capacity (7) of the recipient, and identifying efficient channels for knowledge dissemination and for providing a shared lexicon to all involved (4, 37). In this study, the intervention material (leadership guide/scoring tool/homework/clinical equipment/simulations/measurements) served as a shared lexicon, easing the transfer of knowledge (38). Our findings showed that the dissemination of the material to all staff and further providing a systematic approach to this dissemination were boundaries for the implementation process. Being provided with a single event to learn new knowledge and practices was not perceived as enough to internalise the intervention. Continuity in learning activities is therefore a solution to support the traversing of the syntactic boundary. This is echoed in Greenhalgh et al. (39), stating that continuous access to knowledge is essential for internalisation and adoption of innovations. However, in the context of nursing homes and homecare services characterised by a lack of resources, high workloads, and a high level of sick leave, finding means and arenas to efficiently provide learning is a trade-off to be handled by leaders (40–42).

The semantic boundary included boundaries like ambiguity, lack of perceived impact for practice, and lack of appropriate competence. The semantic boundary was found most related to perceived uncertainty about the facilitator role. The facilitator role is described as important for driving the implementation process (43). However, in order for facilitators to succeed, they need to have appropriate knowledge to be able to take on this role, perceive the intervention as relevant for practice, and possess a thorough understanding of the content they are to teach others. Otherwise, these aspects may end up as boundaries as exemplified in this study. This aspect is also reflected in other research where Lau et al. (19) found that having appropriate knowledge to facilitate provided implementation success, and Cresswell et al. (44) described participants in possession of appropriate knowledge as more open and satisfied about new knowledge and practices. Participants also need to perceive the intervention as relevant for their setting, which is also greatly reflected in existing literature (19, 39, 43–46). It was found important that the facilitators were able to emphasise the inherent relevance in their dissemination of the intervention to their staff. As stated by ter Wal et al. (47), facilitators should be able to pursue the utilisation of external knowledge with a commitment as if it were their own.

Solutions for traversing the semantic boundary are described by Carlile (4) as consisting of means to provide shared understanding. Potential means for obtaining shared understanding are the use of boundary objects or boundary spanners (3, 8, 12, 17, 48, 49). Boundary objects are concrete (e.g., models, pictures) or abstract (e.g., analogies, language, mental models) concepts providing a common point of reference (5, 12, 50). Furthermore, boundary spanners are actors facilitating the transfer of knowledge and information across groups, and as such traversing the semantic boundary (3, 15, 17). This is reflected in this study, where uncertainty of the content, relevance and use acted as barriers to implementation, introducing a need for boundary spanners or boundary objects to form a shared understanding. It is therefore important to ensure time, support and resources for facilitators to obtain an understanding of the intervention before they are to teach others, and to act as a boundary spanner within the organisation.

The pragmatic boundary was found to be related to the change of practices. This aligns with Carlile's (4) definitions of the pragmatic boundary and findings in other studies (2, 5, 10). Changing practices is not an easy task and is often met by resistance (1). Lau et al. (19) describe a culture receptive to change as a key implementation factor. As such, external factors such as a policy of quality improvements in primary care, and incentives such as relevance for practice, should be complemented with the development of a culture that values changes. Resistance is interlinked with the findings of a lack of ownership, feeling unsafe and having different perspectives, even though they take place at the professional level (19, 45). Not believing in their own competences and thus feeling insecure when performing new practices or teaching new knowledge to others, are well described in the self-efficacy literature (51), and furthermore known as essential in individual change theory (45, 52). However, Braithwaite (1) states that behavioural change cannot be fully understood by exploring individual characteristics alone, due to the complexity of the healthcare setting. Perspectives, interests, and incentives may differ across participants and stakeholders, posting a need for trade-offs and converging processes (1, 9). This complexity introduces a challenge for facilitators in deciding on approaches to aligning the implementation process to the situational context and the type of knowledge to be translated into practice (22). Harvey and Kitson emphasise the knowledge boundary framework by Carlile (4) as a means for facilitators to understand knowledge, boundaries and approaches for transferring knowledge across domains in healthcare contexts. Similarly, the knowledge boundary framework was found valuable in this study for taking the type of knowledge to be implemented into account, such as the resistance to changing practices and the challenge of transferring tacit (practice-based knowledge).

Workload is a barrier frequently referred to (19, 43, 45, 53). Carlfjord et al. (54) found workload to reduce engagement in the implementation process. Implementations processes therefore need to take the situational workload into account when initiating interventions, or this it may end up being perceived as a burden for the participants (55). Implementation processes that are not aligned to the situational workload may result in workarounds, thereby not complying with the actual intervention's aim (56). Carlile (4) describes the need for developing shared perspectives, incentives, and interests to be able to traverse the pragmatic boundary. Efforts to converge perspectives, incentives and interests are often time- and resource-demanding (6), and in order for leaders to be efficient, they should strive to develop a culture for change (45).

### 4.1 Strengths and limitations

There are some limitations to this study. First, the qualitative study design, setting and population means that the study findings are not to be perceived as generalisable. New studies should therefore study knowledge boundaries on implementation processes in other settings (e.g., hospital), with other informants (e.g., staff), and for other types of interventions. However, the inclusion of data from both externally and internally driven implementation processes broadens the impact of the findings. Furthermore, the longitudinal research design provides a deeper understanding of the phenomenon. Second, the external and internal case studied were implementing different quality improvement interventions, which can be considered a limitation for the cross-comparison analysis. However, the inclusion of different interventions and implementations processes in the analysis provides an understanding of knowledge boundaries across different approaches which is important for obtaining a more holistic knowledge of the phenomenon. Third, as the external case includes both nursing homes and homecare services, while the internal case only includes homecare services, some differences in the empirical setting are present. Fourth, this study focuses solely on interviews of leaders and professional nurses, and as such other findings might have emerged if interviews with and observations of staff had also been included in the empirical data. However, the focus on leaders across cases provided a more uniform empirical foundation and therefore eased the cross-case analysis. The variety in interventions and setting therefore acts as both a strength and a limitation, yet as this study aims for an explorative focus the variety reveals more of a strength. Fifth, for the external case, the researchers in charge of developing the intervention were also performing the interviews, which might introduce a bias. This bias was not present in the internal case. Strong similarities across cases might, however, indicate that this bias did not introduce major impact. Furthermore, the structure of having the author leading the data analysis not being part of data collection is a strength for the inductive analysis as this structure allows for minimal preconceptions.

The overall strength of this study is the comprehensive dataset, which was acquired over time and included several Norwegian municipalities and sites. The initial aim of developing a new theoretical understanding was changed during the analysis process due to the identified similarities of the findings with the knowledge boundaries framework. However, the use of the knowledge boundaries framework in this study provides novelty as this framework, to the authors knowledge, has not been used to explore implementation of quality interventions in nursing homes and homecare services before.

### 4.2 Conclusion and implications

The aim of this study was to explore and identify knowledge boundaries to the implementation of quality improvement interventions in nursing homes and homecare services. Our inductive findings revealed strong similarities to the knowledge boundaries framework by Carlile (4) and findings were therefore in a later step deductively analysed based on the knowledge boundaries framework. Our findings revealed that the syntactic boundary included the lack of meeting arenas, knowledge transfer and continuity in learning. Furthermore, the syntactic boundary was mostly related to the dissemination and training of staff across the organisation. The semantic boundary consisted of boundaries such as ambiguity, a lack of perceived impact for practice and a lack of appropriate knowledge. This boundary related mostly to uncertainty of the facilitator role. The pragmatic boundary included the lack of ownership, resistance, feeling insecure, workload, different perspectives and a lack of support and focus, all related to change in practices.

Interventions aimed at increasing the quality of healthcare services are a priority for leaders and governments (53, 54, 57). As such, this study has implications for leaders and governments through the understanding of knowledge boundaries that can be expected to be encountered in implementation processes, in both internally driven and externally driven approaches. Furthermore, by relating the identified boundaries to the knowledge boundaries framework of Carlile (4), potential solutions and means to traverse these boundaries are provided. The knowledge boundaries framework by Carlile (4) is a well-known instrument for understanding knowledge boundaries in innovation processes and in cross-disciplinary collaborations. However, the use of this framework for understanding knowledge boundaries in quality improvement processes in nursing homes and homecare services is novel.

At the syntactic boundary, potential solutions include providing meeting arenas and continuity of learning activities. Potential solutions at the semantic boundary include providing facilitators with a deep understanding and ownership of the intervention. At the pragmatic boundary, potential solutions include developing a culture for change. This study also provides theoretical contributions by bridging quality improvement, implementation science and knowledge boundaries in theoretical innovation fields. Finally, the integration of multiple cases, using diverse approaches for implementation, provides empirical understanding from different settings, responding to the call to explore different contextual settings in implementation studies (19, 23, 24).

## Data Availability

The raw data supporting the conclusions of this article will be made available by the authors, without undue reservation.
